# Supplementary material for: Features of DNA–Montmorillonite Binding Visualized by Atomic Force Microscopy
Source: Int J Mol Sci. 2023 Jun 6;24(12):9827. doi: 10.3390/ijms24129827 (PMC10298354; doi:10.3390/ijms24129827)
Supplement: Supplementary file 1 [file ijms-24-09827-s001.zip › ijms-2398990-supplementary.pdf]

Control experiments

DNA+ Graphene oxide

As a control experiment, we performed a study of mechanic binding effect with graphene oxide. Graphene oxide was chosen as another nanomaterial with 2D geometry in order to elucidate the peculiarity of adsorption on montmorillonites. For sample deposition, a 1 µl drop of the 10 mg/l graphene oxide solution in water was placed on the surface of freshly cleaved mica just after that the 1 µl drop of the 10 mM $MgCl_2$ was added and right after that 1 µl drop of the lambda DNA 10 mg/l was added for 1 min. Then, the sample was softly rinsed by placing a 100 µl drop of double distilled water for 1 min and removing it from the surface with a nitrogen flow.

As can be seen in Fig. S1 a few DNA bundles lay on the basal surface of graphene oxide particles.

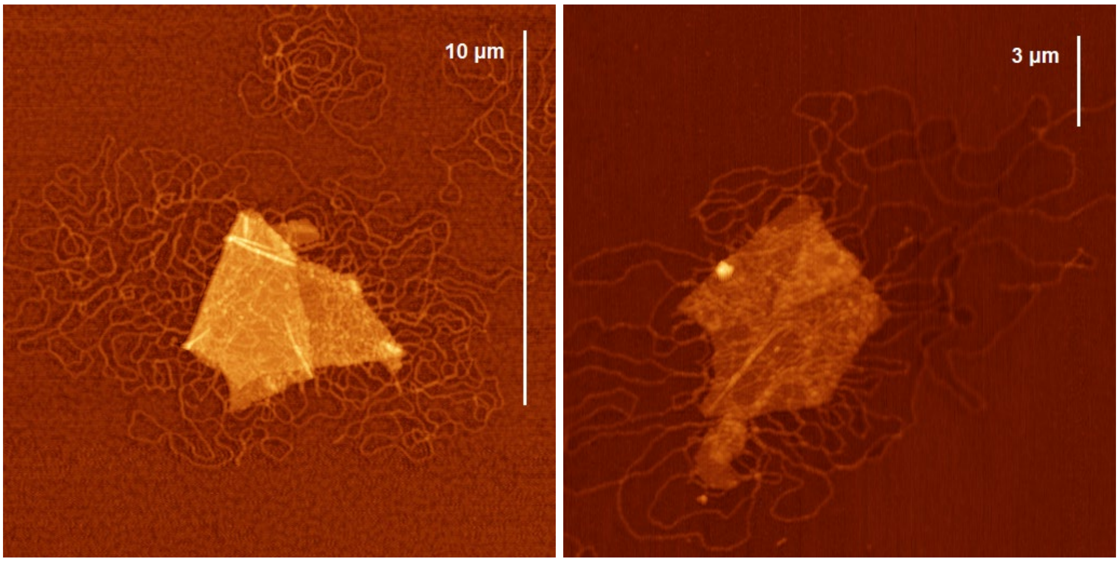

Figure S1. AFM images of the DNA and graphene oxide mixed in solution with $MgCl_2$ and deposited on mica.

DNA and MNT at pH 3.5 and 9.0

Visualization of DNA-Mt complexes at pH 3.5 and pH 9 were performed. It was shown that, at pH 3.5 DNA molecules in such complexes is in the coiled rather than extended state Fig. S2.

At pH 9.0 DNA still mainly binds to the MNT edge joints but quality of AFM is much lower because of large amount of additional salts Fig S3.

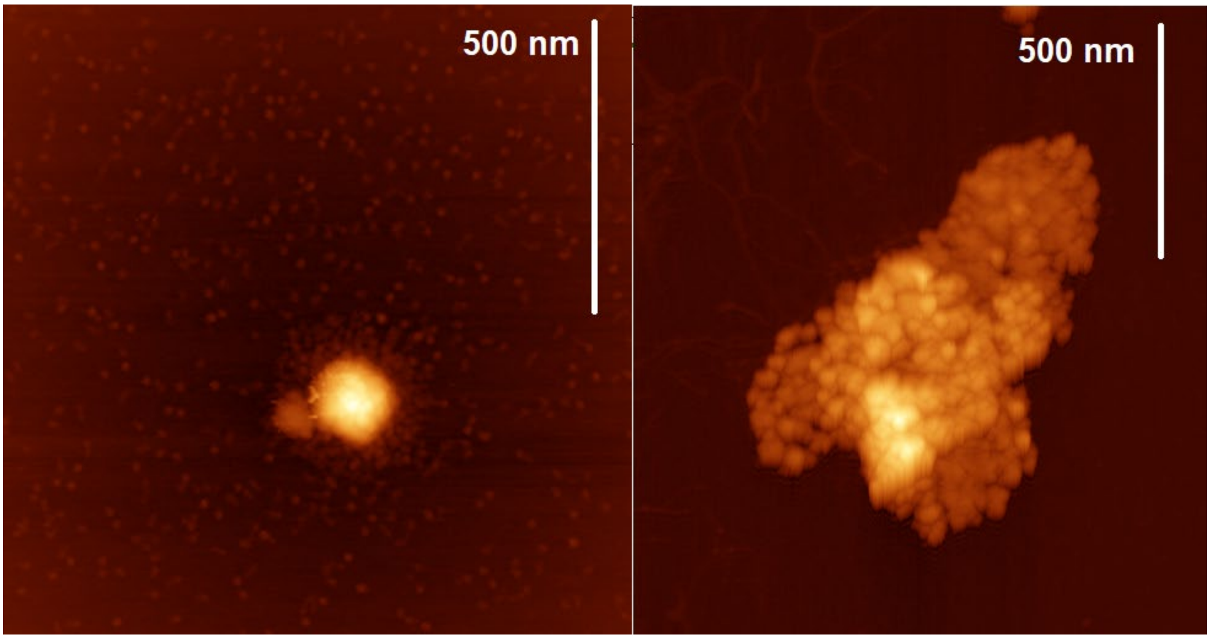

Figure S2. AFM images of the DNA and MNT in solution with pH 3.5 and then deposited on mica.

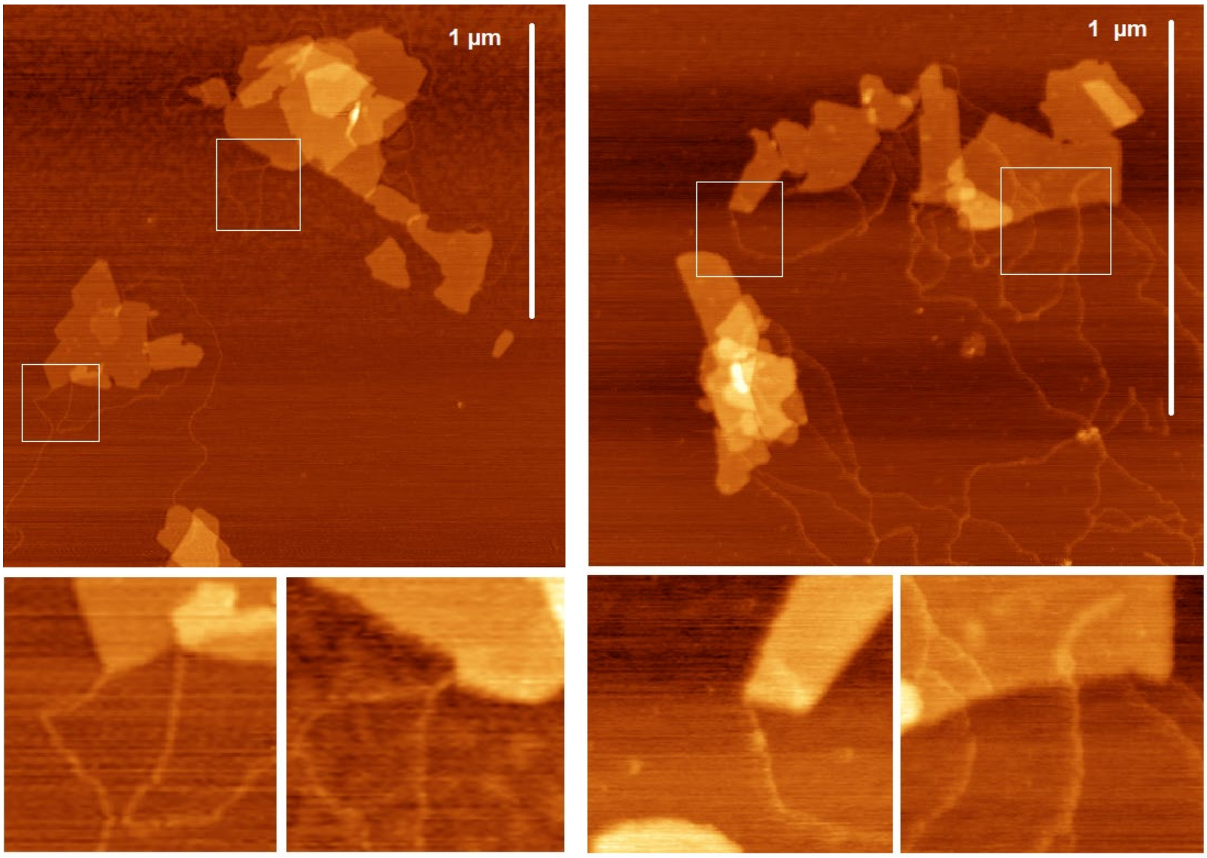

Figure S3. AFM images of the DNA and MNT in 9.0 pH solution deposited on mica.

Gallery of AFM-images of the DNA and MNT mixed in solution with $MgCl_2$ and deposited on mica with closer view of the selected areas.

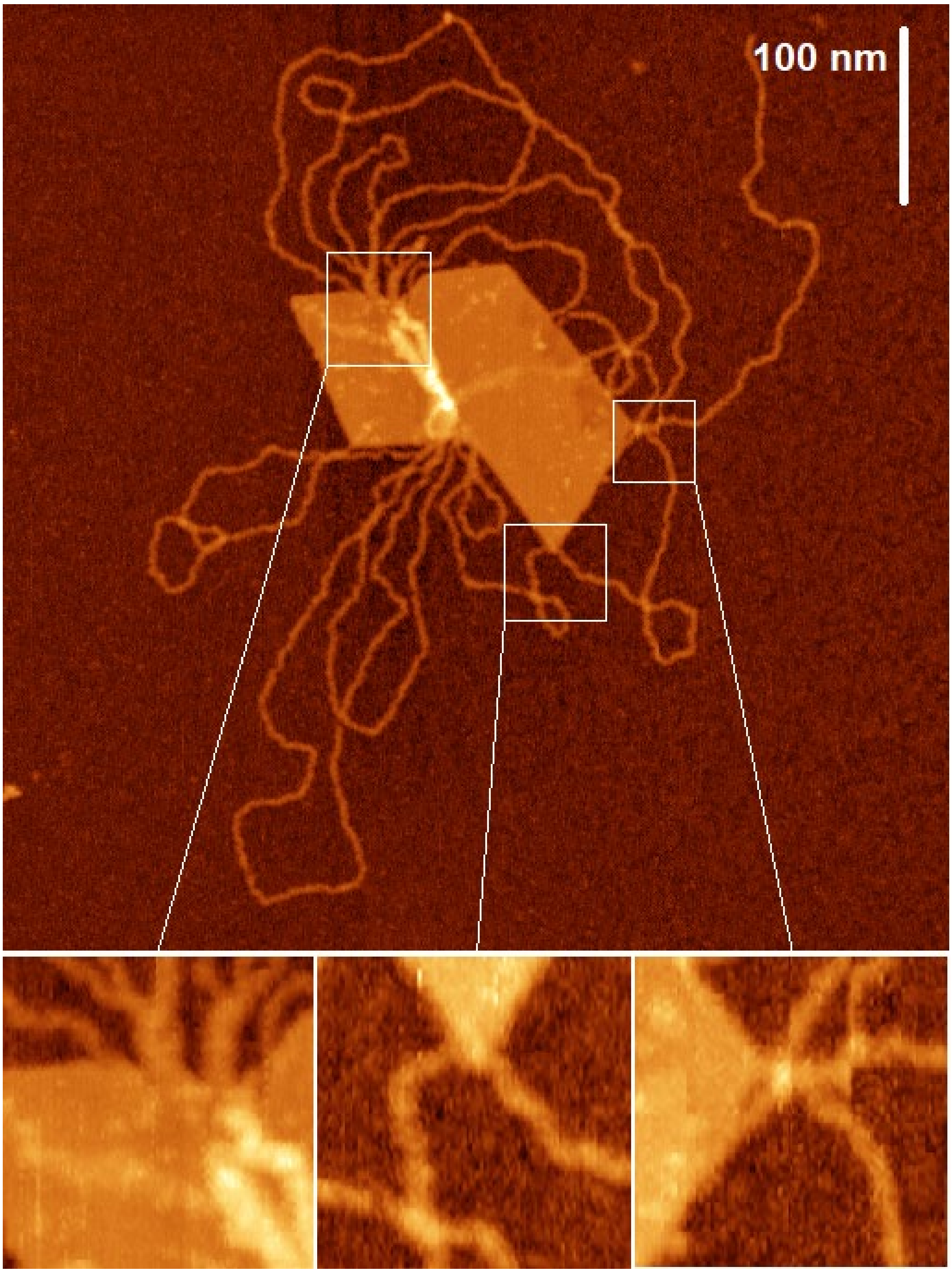

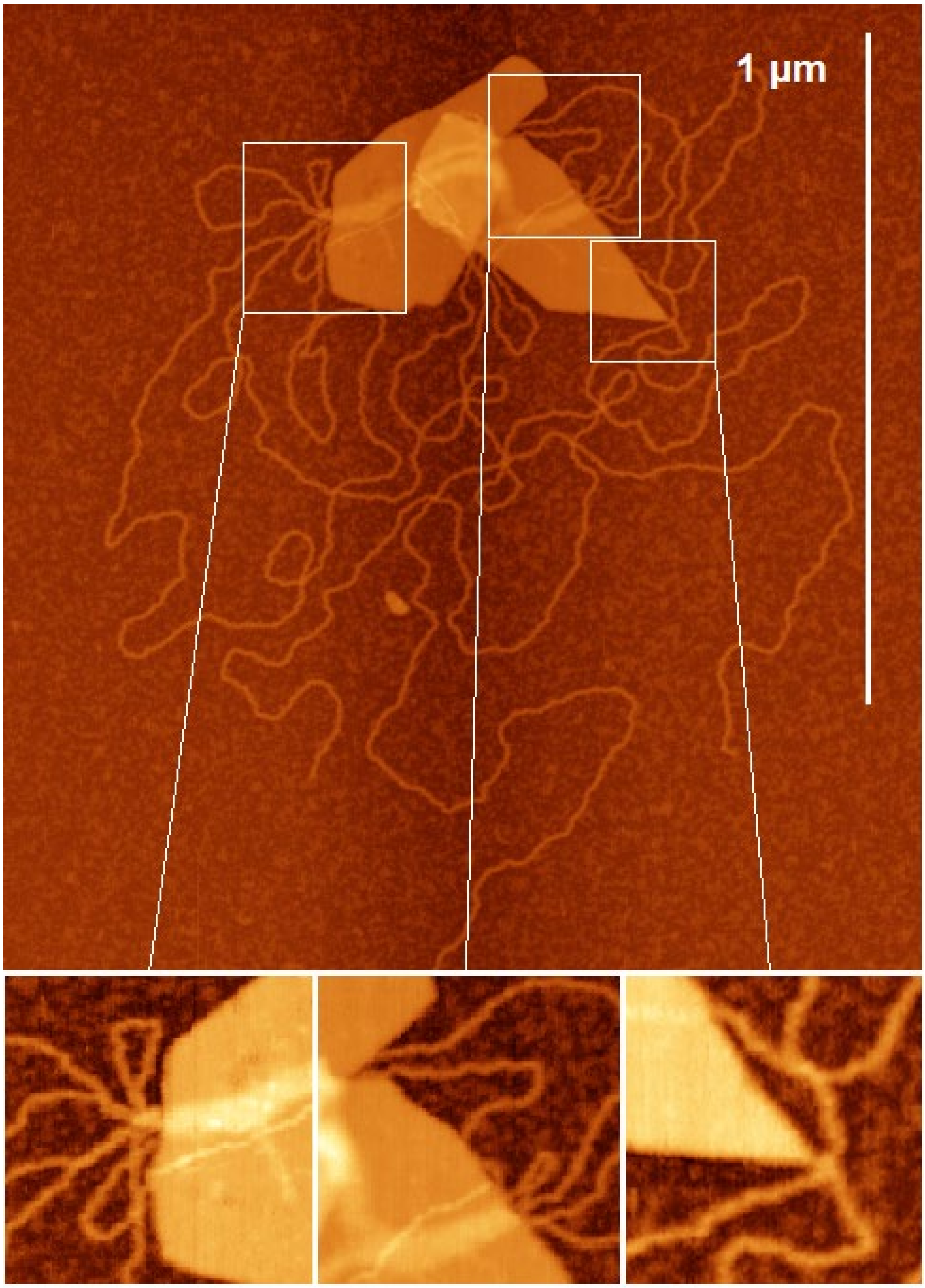
1 µm

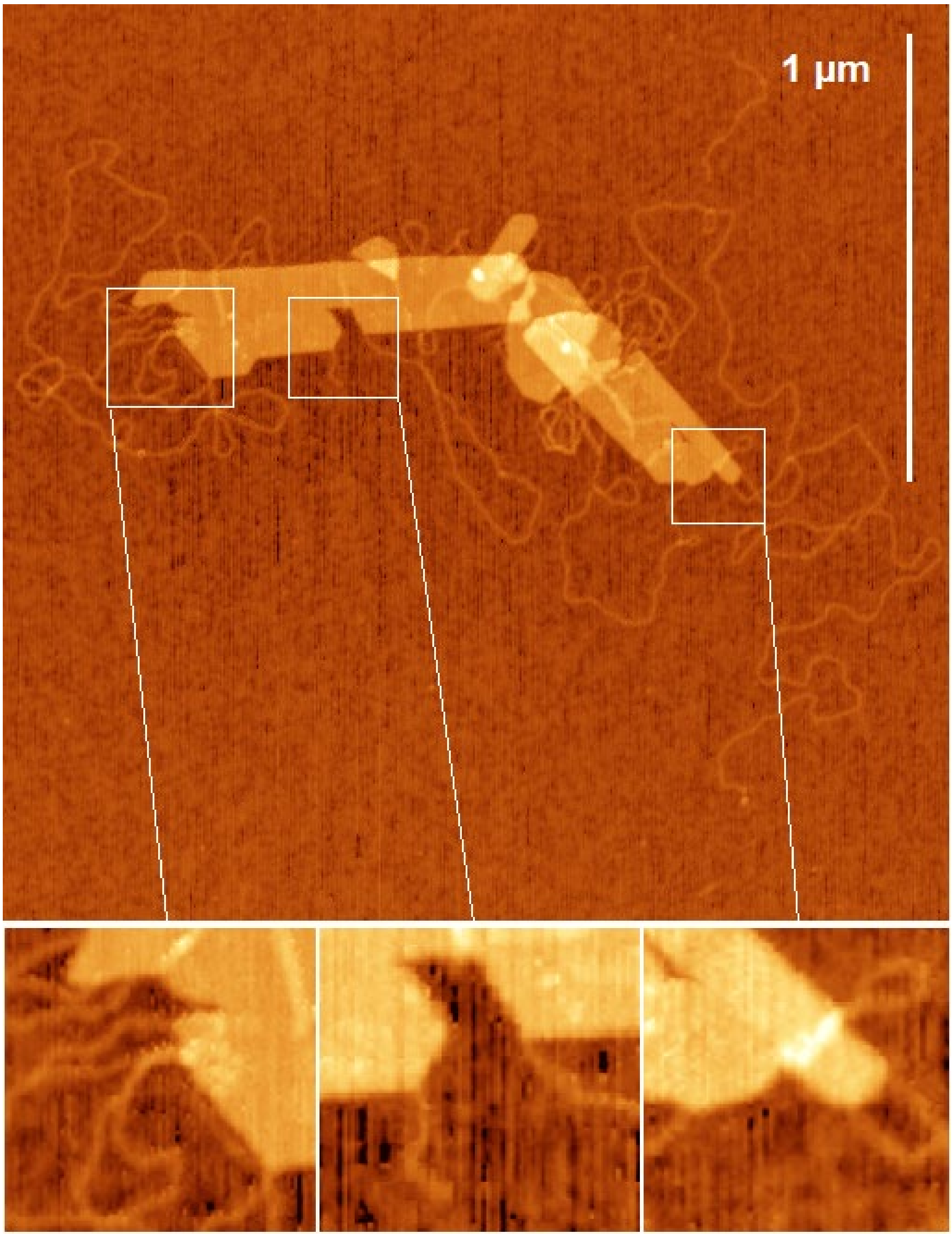

Figure S4. AFM images of the DNA and MNT mixed in solution with $MgCl_2$ and deposited on mica.

Structure of montmorillonite

The periodic bond chain theory [1–6] predicts six preferred edge surfaces for montmorillonite, namely, (010), (100), (±110), and (±130). The edge surfaces could be obtained if the crystal is cut in a way that preserves the chains of Si tetrahedra and Al octahedra which make up the structure. However, in the case of a simplified orthogonal unit cell only four of the corresponding surfaces are different from each other, namely, (010), (100), (110), and (130). The surfaces (130) and (-130) and (110) and (-110) are very similar in general and identical in case of orthogonality. For montmorillonite (010) and (110) edge surfaces were found to be more abundant [6]. Thus, we inspected joints formed by these two edge surfaces.

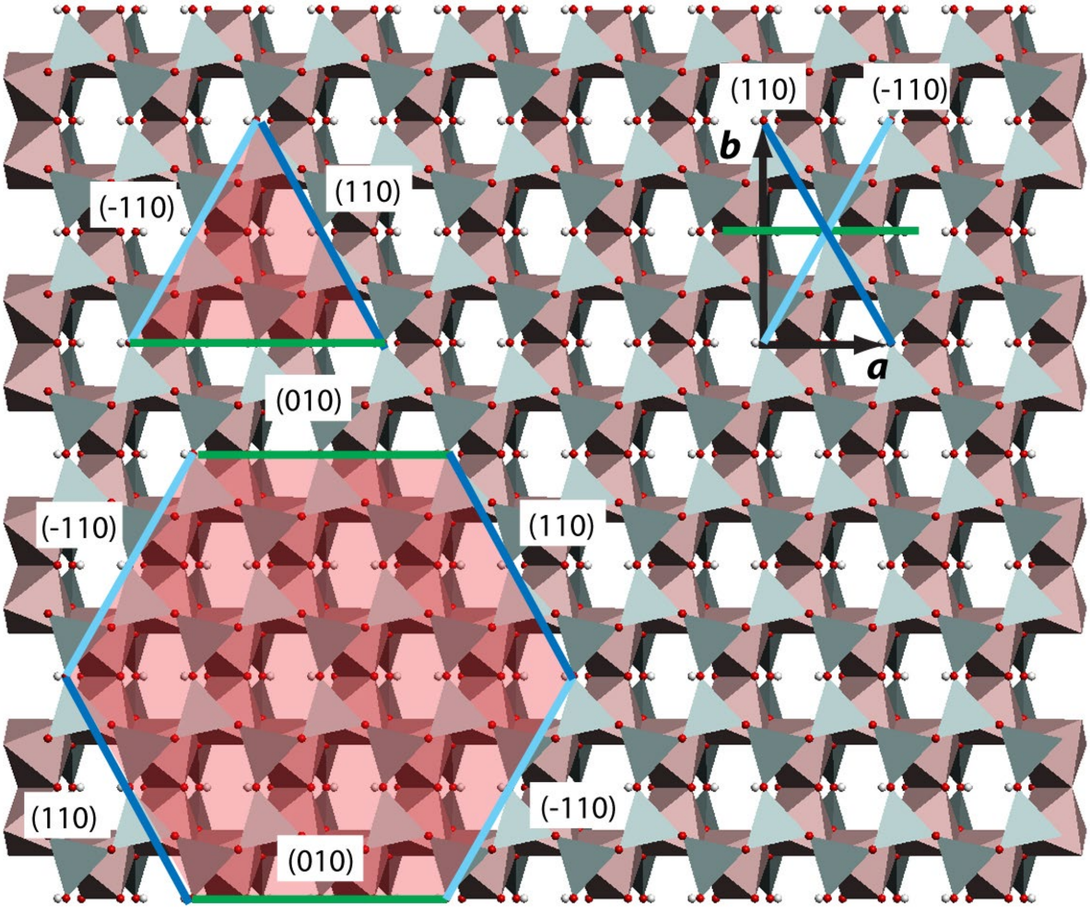

Figure S5. Top view of a montmorillonite layer from direction [001]. Si tetrahedra are grey, Al octahedra are brown. The unit cell vectors ***a*** and ***b*** are shown by black arrows. Green, light blue, and dark blue lines indicate the edge orientations (010), (-110), and (110), respectively.

Fig. S5 shows top view of montmorillonite and three edge orientations, horizontal green line corresponds to (010) orientation, light blue and blue lines correspond to (-110) and (110) orientations, respectively. Cutting montmorillonite bulk in these directions would result in various shapes of particles. We consider a triangular particle that exhibits joints between (010) and (110) and between (110) and (110) edge surfaces, see Fig. S5. Both pairs of edges join at 60º. In turn, in hexagon-shaped particle the pairs of edges join at 120º. To note, in the main text, only half of the hexagonal particle is shown.

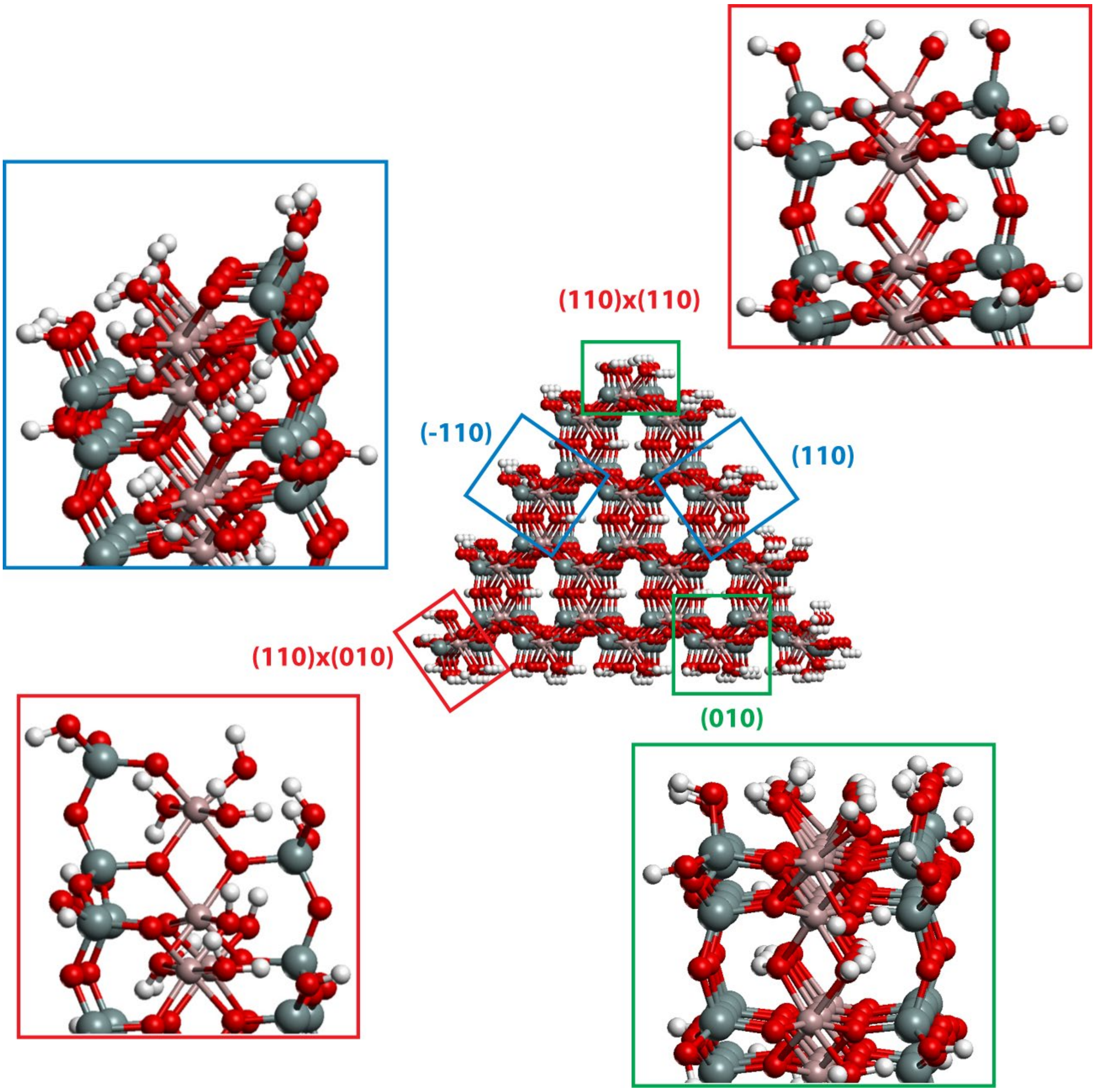

Figure S6. Top view of the neutral triangular shape. The squares show the fragments of (110), in blue, and (010), in green, edge surfaces, as well as (110)×(110) and (110)×(010) edge joints in red.

We used a triangular shape (periodic in ***c*** direction perpendicular to the paper plane) in order to compare various Al centers with respect to their affinity to OH_2 ligands. When DNA adsorbs on montmorillonite particle with its phosphate group, phosphate substitutes OH or OH_2 ligands of the surface Al center. Thus, the number of such dangling ligands and their affinity to Al center should give us qualitative estimate of how reactive is particular Al center. For simplicity, a triangular particle was cut from the bulk without any charged substitutions and without interlayer cations. The dangling bonds were saturated by protons and OH groups to preserve the neutrality of the system. The top view of the neutral triangle shape is shown in the center of Fig. S6. Small squares in Fig. S6 show the fragments of (110) and (010) edge surfaces, as well as (110)×(110) and (110)×(010) edge joints.

We used the following equation to estimate the OH_2 dissociation energy:

$$MNT_{CN(Al)=6}\text{-}OH_2 \rightarrow MNT_{CN(Al)=5} + H_2O \qquad (1)$$

We carried out first-principles density functional calculations using the plane-wave based Vienna *ab initio* simulation package (VASP) [7–10]. The generalized gradient approximation (GGA) to the exchange-correlation potential was applied as parameterized by Perdew, Becke, and Ernzerhof (PBE) [11,12] for the structure optimization. The effect of core electrons was accounted for by the projector-augmented wave (PAW) method, as implemented in VASP [13,14]. When optimizing the unit cell, we adopted an energy cut-off of 400 eV. Integrations over the Brillouin zone were carried out using the Γ-point only. During the self-consistency cycles, the total energy was converged to 10^{-5} eV. For geometry optimizations, forces acting on ions were required to be less than 2×10^{-4} eV/pm. The program VASP provides compensating corrections for charged unit cells of cubic lattices only. Therefore, we employed only neutral unit cells for our calculations.

In order to estimate the solvation corrections in Eq. (1), we used cluster of one layer of the triangular shape, so called one-layer triangle particle. We used the software Turbomole, version 6.6 [15,16]. The free energy of solvation ΔG_{solv} was calculated by means of single point calculations using the polarized continuum solvation model COSMO as implemented in Turbomole [17]. In these calculations, we employed the same GGA functional PBE [11]. To accelerate the DFT calculations, we applied the resolution-of-identity (RI-J) approximation for evaluating the Coulomb term of the electron-electron interaction, together with a suitable auxiliary basis set [18]. We represented the Kohn-Sham orbitals with standard double-zeta basis sets [19]. In the self-consistent field procedure to determine the electronic structure, the total energy was converged to 10^{-6} au.

Results of these calculations are shown in Table S1.

Table S1. Energetical characteristics calculated according to Eq. (1) in gas phase and in solution, all values are in kcal/mol.

	E(1)gas	E(1)sol
(010)	9.7	10.6
(110)	14.0	16.0
(110)×(110)	5.1	3.6
(110)×(010)	4.0	2.2

PCR experiment

Sorption and desorption of total nucleic acids including both RNA and DNA from mouse blood serum were compared for MNT and conventional silica particles according to [20]. Reverse transcription with subsequent real-time PCR with TaqMan probe (Fig. S7) revealed similar threshold cycles (Ct) for nucleic acids isolated by using silica particles (Ct =31.4) and MNT (Ct =32.8) that suggested approximately equal amounts of RNA desorbed from both sorbents.

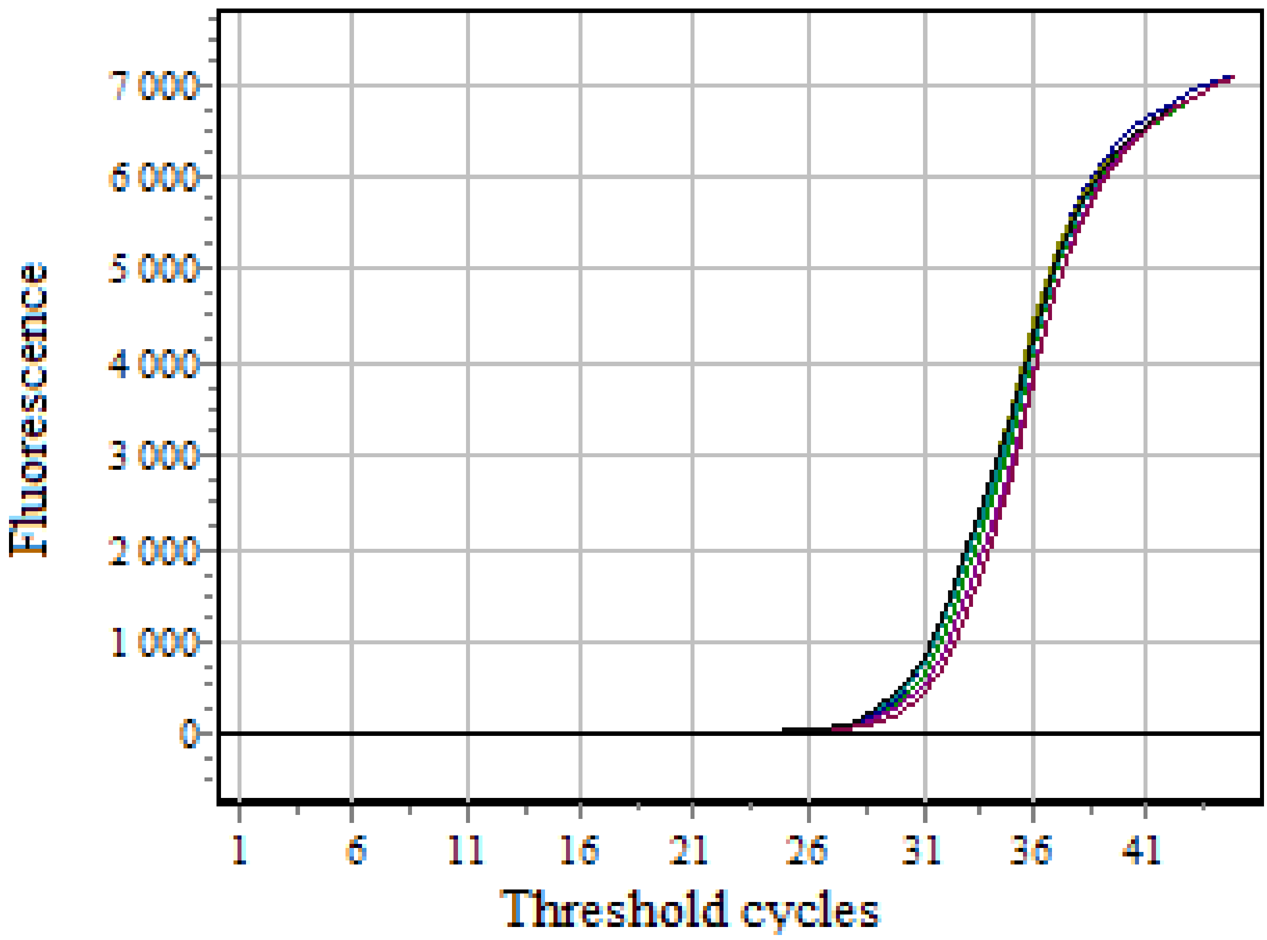

Figure S7. Results of reverse transcription with real-time PCR of mouse actin RNA isolated from blood serum using silica particles (green curve) and MNT (purple curve). Horizontal black line corresponds to threshold.

Based on AFM images (Fig. 1-3) and RT^2-PCR data (Fig. S7) sorption of nucleic acids onto MNT surfaces could take place in 10 mM $MgCl_2$ water solution and in chaotropic agent – 5.5 M guanidinium thiocyanate [20]. Desorption of nucleic acids was efficient in water solution at elevated temperatures 65^0C for 5-10 min in a broad pH range from 5 to 8.

Literature

1. Hartman, P.; Perdok, W.G. On the Relations between Structure and Morphology of Crystals. II. *Acta Cryst* **1955**, *8*, 521–524, doi:10.1107/S0365110X55001679.
2. Hartman, P.; Perdok, W.G. On the Relations between Structure and Morphology of Crystals. III. *Acta Cryst* **1955**, *8*, 525–529, doi:10.1107/S0365110X55001680.
3. Hartman, P.; Perdok, W.G.; IUCr On the Relations between Structure and Morphology of Crystals. I Available online: http://scripts.iucr.org/cgi-bin/paper?S0365110X55000121 (accessed on 14 November 2019).

4. White, G.N.; Zelazny, L.W. Analysis and Implications of the Edge Structure of Dioctahedral Phyllosilicates. *Clays Clay Miner.* **1988**, *36*, 141–146, doi:10.1346/CCMN.1988.0360207.
5. Kuwahara, Y. In-Situ AFM Study of Smectite Dissolution under Alkaline Conditions at Room Temperature. *American Mineralogist* **2006**, *91*, 1142–1149, doi:10.2138/am.2006.2078.
6. Kraevsky, S.V.; Tournassat, C.; Vayer, M.; Warmont, F.; Grangeon, S.; Ngouana Wakou, B.F.; Kalinichev, A.G. Identification of Montmorillonite Particle Edge Orientations by Atomic-Force Microscopy. *Applied Clay Science* **2020**, *186*, 105442, doi:10.1016/j.clay.2020.105442.
7. Kresse, G.; Hafner, J. Ab Initio Molecular Dynamics for Liquid Metals. *Phys. Rev. B* **1993**, *47*, 558–561, doi:10.1103/PhysRevB.47.558.
8. Kresse, G.; Hafner, J. Ab Initio Molecular-Dynamics Simulation of the Liquid-Metal--Amorphous-Semiconductor Transition in Germanium. *Phys. Rev. B* **1994**, *49*, 14251–14269, doi:10.1103/PhysRevB.49.14251.
9. Kresse, G.; Furthmüller, J. Efficiency of Ab-Initio Total Energy Calculations for Metals and Semiconductors Using a Plane-Wave Basis Set. *Computational Materials Science* **1996**, *6*, 15–50, doi:10.1016/0927-0256(96)00008-0.
10. Kresse, G.; Furthmüller, J. Efficient Iterative Schemes for Ab Initio Total-Energy Calculations Using a Plane-Wave Basis Set. *Phys. Rev. B* **1996**, *54*, 11169–11186, doi:10.1103/PhysRevB.54.11169.
11. Perdew, J.P.; Burke, K.; Ernzerhof, M. Generalized Gradient Approximation Made Simple. *Phys. Rev. Lett.* **1996**, *77*, 3865–3868, doi:10.1103/PhysRevLett.77.3865.
12. Perdew, J.P.; Burke, K.; Ernzerhof, M. Generalized Gradient Approximation Made Simple [Phys. Rev. Lett. 77, 3865 (1996)]. *Phys. Rev. Lett.* **1997**, *78*, 1396–1396, doi:10.1103/PhysRevLett.78.1396.
13. Blöchl, P.E. Projector Augmented-Wave Method. *Phys. Rev. B* **1994**, *50*, 17953–17979, doi:10.1103/PhysRevB.50.17953.
14. Kresse, G.; Joubert, D. From Ultrasoft Pseudopotentials to the Projector Augmented-Wave Method. *Phys. Rev. B* **1999**, *59*, 1758–1775, doi:10.1103/PhysRevB.59.1758.
15. Ahlrichs, R.; Bär, M.; Häser, M.; Horn, H.; Kölmel, C. Electronic Structure Calculations on Workstation Computers: The Program System Turbomole. *Chemical Physics Letters* **1989**, *162*, 165–169, doi:10.1016/0009-2614(89)85118-8.
16. Treutler, O.; Ahlrichs, R. Efficient Molecular Numerical Integration Schemes. *J. Chem. Phys.* **1995**, *102*, 346–354, doi:10.1063/1.469408.
17. Klamt, A.; Schüürmann, G. COSMO: A New Approach to Dielectric Screening in Solvents with Explicit Expressions for the Screening Energy and Its Gradient. *J. Chem. Soc., Perkin Trans. 2* **1993**, 799–805, doi:10.1039/P29930000799.
18. Eichkorn, K.; Treutler, O.; Öhm, H.; Häser, M.; Ahlrichs, R. Auxiliary Basis Sets to Approximate Coulomb Potentials. *Chemical Physics Letters* **1995**, *240*, 283–290, doi:10.1016/0009-2614(95)00621-A.
19. Schäfer, A.; Horn, H.; Ahlrichs, R. Fully Optimized Contracted Gaussian Basis Sets for Atoms Li to Kr. *J. Chem. Phys.* **1992**, *97*, 2571–2577, doi:10.1063/1.463096.
20. Boom, R.; Sol, C.J.; Salimans, M.M.; Jansen, C.L.; Dillen, P.M.W.; Noordaa, J. van der Rapid and Simple Method for Purification of Nucleic Acids. *Journal of Clinical Microbiology* **1990**, *28*, 495–503.
